# Utility of clinical features with fine needle aspiration biopsy for diagnosis of Warthin tumor

**DOI:** 10.1186/s40463-019-0366-3

**Published:** 2019-08-29

**Authors:** Thomas So, Axel Sahovaler, Anthony Nichols, Kevin Fung, John Yoo, Michele M. Weir, S. Danielle MacNeil

**Affiliations:** 1 Schulich School of Medicine and Dentistry, Western University, London, ON Canada; 2 Department of Otolaryngology-Head and Neck Surgery, Western University and London Health Sciences Centre, London, ON Canada; 3 Department of Pathology and Laboratory Medicine, Western University and London Health Sciences Centre, London, ON Canada; 4 Victoria Hospital, Suite B3-429, 800 Commissioners Rd E, 31, London, ON N6A 5W9 Canada

**Keywords:** Warthin tumor, Fine needle aspiration biopsy, Resection, Sensitivity, Positive predictive value, Clinical features

## Abstract

**Background:**

Conservative management of Warthin tumor (WT) may be a viable alternative to surgery, but there are concerns of missed malignancies on fine needle aspiration biopsy (FNAB). The purpose of this study is to measure the sensitivity and positive predictive value of FNAB for WT, and to identify clinical features associated with WT that can aid in this diagnosis.

**Methods:**

Retrospective analysis of patients from January 1, 2006 to April 30, 2017 at a tertiary care center in London, Ontario, Canada. All patients with a diagnosis of WT on FNAB or resection were included. Electronic medical records were identified for 177 patients that fit the criteria. Study outcomes included the sensitivity and positive predictive value of FNAB alone for WT, and, when including clinical features associated with WT.

**Results:**

The mean age of patients in this study was 63.2 years (SD 10.4); 115 (65%) were male, and 157 (89%) were past or present smokers. The measured sensitivity and positive predictive value of FNAB for WT were 95.8 and 97.2% respectively. Two cases were classified as WT on FNAB but confirmed at resection as mucoepidermoid carcinoma and acinic cell carcinoma. When only patients with multifocal, bilateral or incidental tumors were assessed, sensitivities and positive predictive values for each were 100%. Isolating for inferior pole location also resulted in a positive predictive value of 100%.

**Conclusions:**

The sensitivity and positive predictive value of FNAB for WT in this study are high, with two false negatives on FNAB. Multifocal, bilateral, incidentaloma and inferior pole location were identified as potential clinical features that may increase the diagnostic confidence for WT, strengthening the argument for conservative management in these patients. Overall, this study serves as an initial exploration into whether clinical features may be included with FNAB results to improve the sensitivity and positive predictive value of diagnosing WT. Further research is necessary before these findings can be translated into clinical practice.

## Background

Warthin tumor (WT), the second most common benign tumor of the parotid gland, makes up approximately 15% of all parotid tumors [1]. The treatment of choice is superficial parotidectomy [2, 3], but this may be associated with complications including both temporary and permanent facial nerve injury, Frey’s syndrome, and hematoma [4, 5]. WT has a slow growth rate, is typically asymptomatic, is more common in smokers, is rarely associated with malignancy, and generally occurs in patients in their 60s [2, 6]. In recent years, more patients are diagnosed with WT as an incidental finding on PET scan. Given that many of these patients are older, usually smokers with other comorbidities and the tumor is found incidentally, conservative management is an attractive alternative to surgery [3, 4, 7]. Literature on the safety of observation for patients with WT is sparse [3].

In addition to the paucity of research in this area, conservative management has not been widely adopted due to the uncertainty of the pre-operative diagnosis in the form of fine needle aspiration biopsy (FNAB) [3, 4, 7]. FNAB is minimally invasive and cost-effective [4], but suffers from low sensitivity, specificity and accuracy [7, 8]. This is compounded by the problem that the FNAB appearance of WT may sometimes overlap with other tumors, some of which are malignant [8]. Thus, confirming the diagnosis with resection is important because of these possible missed malignancies.

Several clinical features have been associated with WT, namely advanced age, smoking, and the male sex [4, 7, 9, 10]. WT is also frequently seen as multifocal, bilateral, and located in the inferior pole of the parotid [9]. In addition, it is not uncommon for WT to be discovered as an incidentaloma on imaging [11–13]. Although many papers have reported these associations, there has not been a study to our knowledge that has examined if these features could aid in the diagnosis of WT.

In the present investigation, we analyzed the pathology data of patients with parotid tumors who have received both FNAB and resection confirmation of the diagnosis to determine the sensitivity and positive predictive value (PPV) of FNAB for WT. We then included the clinical features of these patients into our analysis to determine if any of the features associated with WT improve the sensitivity and positive predictive value of the diagnosis.

## Methods

A retrospective analysis was conducted on all patients with a diagnosis of WT on FNAB or resection presenting to the London Health Sciences Centre (LHSC) Department of Otolaryngology-Head and Neck Surgery from January 1, 2006 to April 30, 2017. Patients who met these criteria were identified through a search of the electronic pathology database. The electronic medical records of these patients were accessed, which contain all information relating to imaging, pathology, procedures and clinic reports. Data from these records were collected, including the FNAB diagnosis, resection diagnosis, age, gender, smoking history, symptoms, location of the tumor in the parotid, and if the tumor was multifocal, bilateral or an incidentaloma.

Besides the resection diagnosis, only information known at presentation to clinic or on initial workup was collected, in keeping with the goal of this study. Age was defined as the age at presentation and patients were classified as smokers if they had any history of smoking. Only symptoms noted at initial presentation were recorded, any symptoms that developed later were not. Similarly, the tumor was recorded as multifocal or bilateral only if discovered on initial imaging workup. Patients with a diagnosis of WT or a history of parotidectomy on the contralateral side prior to January 1, 2006 were also considered bilateral. The inferior pole of the parotid was defined as synonymous with ‘parotid tail’, ‘angle of the mandible’ and ‘lower parotid’. The tumor was considered an incidentaloma if the tumor was discovered on any imaging modality including PET, CT, MRI and ultrasound performed for an unrelated medical condition or symptom.

The FNAB diagnoses were classified as neoplasm consistent with Warthin tumor (WT), negative for malignancy (not WT), or indeterminate. Phrases such as ‘favours Warthin,’ ‘suggestive of Warthin,’ ‘consistent with Warthin,’ and ‘suspicious for Warthin’ were considered a diagnosis of WT. An indeterminate diagnosis was considered to include phrases such as ‘oncocytic neoplasm,’ ‘differential includes Warthin,’ ‘cystic contents,’ and ‘insufficient material for diagnosis.’ Diagnoses classified as negative for malignancy (not WT) also used the phrases ‘favours,’ ‘suggestive of,’ consistent with’ and ‘suspicious for,’ but identified a tumor other than WT.

There was no clinical record of smoking status for 12 (6.8%) patients and 14 (7.9%) patients had no record of whether the tumor was located in the inferior pole. These patients were assumed to be negative with relation to the clinical feature being studied, to prevent inflation of our results. For example, they were assumed to be non-smokers or the tumor was assumed to be in the body of the parotid.

The collected data was used to calculate the sensitivity and PPV of FNAB for WT at our institution. Sensitivity was defined as the number of patients with both FNAB and resection diagnoses of WT, over the number of patients with a resection diagnosis of WT and a FNAB diagnosis of either WT or not WT. Similarly, PPV was defined as the number of patients with both FNAB and resection diagnoses of WT, over the number of patients with a FNAB diagnosis of WT and a resection diagnosis of either WT or not WT. This study was approved by Western University’s Health Sciences Research Ethics Board and Lawson Health Research Institute.

## Results

Electronic medical records were present for 177 patients with a diagnosis of WT on FNAB or resection from January 1, 2006 to April 30, 2017 at LHSC. The mean age of these patients was 63.2 years (SD 10.4); 115 (65%) of them were male, and 157 (89%) had smoked sometime in their life. Of the 177 cases reviewed, 127 (72%) of the patients presented asymptomatically, 145 (82%) of the tumors were in the inferior pole of the parotid, and 28 (16%) were incidentalomas found on imaging. On initial workup, 36 (20%) of the cases were found to be multifocal, and 36 (20%) were bilateral. The patients were then subdivided based on their FNAB and resection diagnoses, and their clinical characteristics (Table 1). One-hundred and twenty-five patients had histopathological diagnoses of WT; the corresponding FNAB diagnoses were as follows: 69 (55.2%) were diagnosed with WT, 3 (2.4%) were diagnosed with not WT, 28 (22.4%) were indeterminate, and 25 (20%) had no record of diagnosis on FNAB.

**Table 1:** Patient characteristics based on their FNAB and resection diagnoses

FNAB Diagnosis	Resection Diagnosis	No.	Age, mean (SD)^a^	Male (%)	Smoker (%)^b^	Asymptomatic (%)^a^	Inferior Pole (%)	Multifocal (%)^c^	Bilateral (%)^c^	Incidentaloma (%)
Warthin	Warthin	69	62.0 (9.6)	50 (72)	65 (94)	48 (70)	56 (81)	18 (26)	18 (26)	10 (14)
Warthin	No record	50	68.1 (10.9)	29 (58)	43 (86)	39 (78)	41 (82)	6 (12)	9 (18)	15 (30)
Warthin	Not Warthin	2	54.5 (9.2)	1 (50)	1 (50)	1 (50)	0 (0)	0 (0)	0 (0)	0 (0)
Not Warthin	Warthin	3	55.0 (14.1)	2 (67)	3 (100)	3 (100)	2 (67)	0 (0)	0 (0)	0 (0)
Indeterminate	Warthin	28	61.5 (9.8)	15 (54)	24 (86)	18 (64)	24 (86)	6 (21)	4 (14)	2 (7)
No record	Warthin	25	60.2 (9.2)	18 (72)	21 (84)	18 (72)	22 (88)	6 (24)	5 (20)	1 (4)

^a^Measured at presentation

^b^Any history of smoking was included

^c^Tumor characteristic on initial workup

Using the subgroups created, the sensitivity and PPV of FNAB for WT at our institution were calculated. There were 69 patients with WT diagnoses on both FNAB and resection, and 3 patients with a FNAB favouring not WT but a resection identifying WT, resulting in a sensitivity of 95.8%. Of the 3 patients with FNAB not classified as WT, the FNAB diagnoses were: chronic sialadenitis, squamoid neoplasm, and mucinous cystic lesion. There were also 2 patients with a FNAB diagnosis of WT but a resection without WT, leading to a PPV of 97.2%. In these cases, the tumors were classified as mucoepidermoid carcinoma and acinic cell carcinoma in the resection specimen.

To identify clinical features of interest, the sensitivity and PPV of FNAB for WT after isolating for the various features were calculated (Table 2). Of note, sensitivity and PPV were found to be 100% when using FNAB to diagnose WT for tumors that were multifocal, bilateral or found as an incidentaloma. PPV was also 100% for tumors that were diagnosed as WT on FNAB that were located in the inferior pole of the parotid.

**Table 2:** Sensitivity and PPV of FNAB for WT after isolating for various clinical features

Clinical feature	Sensitivity (%)	PPV (%)
Age 60+ (*n* = 42)^a^	97.6	97.6
Male (*n* = 53)	96.2	98.0
Smoker (*n* = 69)^b^	95.6	98.5
Asymptomatic (*n* = 52)^a^	94.1	98.0
Inferior pole (*n* = 58)	96.6	100
Multifocal (*n* = 18)^c^	100	100
Bilateral (*n* = 18)^c^	100	100
Incidentaloma (*n* = 10)	100	100

^a^Measured at presentation

^b^Any history of smoking was included

^c^Tumor characteristic on initial workup

## Discussion

The data demonstrates that several of the clinical features associated with WT investigated in this study (multifocal, bilateral, incidentaloma and inferior pole location) can improve our confidence in a FNAB diagnosis favoring WT. In this study, when the FNAB was consistent with WT, WT was identified on final histology in all patients when the tumor was multifocal, bilateral or found as an incidentaloma. Also, when the tumor had any one of these three features or was in the inferior pole of the parotid, then all patients in this study with a FNAB diagnosis favoring WT were found to have WT at resection.

Comparing the overall sensitivity and PPV of FNAB for WT at our institution with values in the literature (Table 3), it appears that LHSC outcomes are comparable or better than other institutions [4, 7, 8, 10, 14]. This may be related to cytopathology specialization at LHSC, the increased experience with WT and pitfalls in diagnoses of salivary gland tumours on FNAB, and the strict criteria used for WT diagnosis on FNAB. Despite the high PPV found however, two tumors initially classified as WT in our study were malignant at resection, highlighting one of the reasons why many surgeons still recommend performing a parotidectomy. Other studies have also found that the majority of the cases misdiagnosed as WT turn out to be malignant [4], which may be why most research to date on the management of WT only examine various extents of surgery without including conservative management [15, 16]. Acinic cell carcinoma and mucoepidermoid carcinoma are well-known pitfalls in the diagnosis of WT on FNAB [17–19].

**Table 3:** Comparison of sensitivity and PPV of FNAB for WT among the literature

Study	Sensitivity (%)	PPV (%)
Current study (*n* = 74)	95.8	97.2
Parwani and Ali (*n* = 27)^14^	74	–
Kim et al. (*n* = 21)^10^	85.7	–
Jeong et al. (*n* = 35)^8^	76.5	96.3
Veder et al. (*n* = 133)^4^	–	95.5
Vlantis et al. (*n* = 40)^7^	–	95

To the best of our knowledge, this is the first study to examine the inclusion of clinical features with the FNAB diagnosis of WT to determine the possibility of conservative management. An increasing number of these tumors are being diagnosed [2, 20], and this increase is potentially linked to the increased use of imaging in health care. In our study, 28 (16%) of patients were unaware that they had a parotid tumor until it was incidentally noted on imaging done for an unrelated medical condition. Given that WT is often asymptomatic, visually unnoticeable, slow growing and rarely associated with malignancy [2, 6], conservatively managing the WT is an attractive alternative to surgery. In our study, 50 (41%) of patients with a FNAB diagnosis of WT had no record of resection. This may be due to the patient choosing conservative management, not being fit for surgery, or undergoing surgery at another center. This proportion seems slightly higher at other institutions. Vlantis et al. reported that 36 (47%) of their 76 WT cases were treated conservatively [7]. Veder et al. reported that conservative treatment was done for 177 (57%) of their 310 WT cases [4]. In spite of our findings indicating that there is a high sensitivity and PPV of WT in patients with clinical features suggestive of WT and an FNAB indicating WT, we hesitate to recommend observation given our small sample size. However, we acknowledge that some clinicians will opt for observation in medically unfit patients following careful discussion with the patient. The long-term outcomes of this group of patients with WT who undergo observation requires further investigation.

Strengths of our study include the overall generalizability of the results, as our patient characteristics are in concordance with the literature [4, 7, 9, 10]. We limited our sample to FNAB and final histology from a tertiary care centre with pathologists experienced in salivary gland cytology and histology, limiting the heterogeneity of pathologic findings. This study also examined the vast majority of clinical features associated with WT highlighted in the literature [4, 7, 9–13]. The ease of access to imaging at our tertiary care centre allowed us to more accurately label tumors as multifocal, bilateral or in the inferior pole of the parotid. Furthermore, the concrete definitions used for the clinical features in the methodology allows for better comparisons to be made with past and future studies.

One of the limitations of this study is that the patients managed conservatively could not be included in the sensitivity and PPV calculations. There are numerous reasons as to why a patient might not have undergone surgery, one of which could be the benign clinical presentation of the lesion. This would suggest that the sensitivity and PPV measured in this study would not be adversely affected if resection results were obtained for all patients. Another limitation is that the heterogeneous categorization of FNAB diagnoses were not fully captured. Phrases expressing various degrees of certainty with regards to the FNAB diagnosis of WT were grouped together without risk stratification. There were also a large number of patients (*n* = 56) who did not have a FNAB available (*n* = 25) or were classified as ‘Not Warthin’ (*n* = 3) or ‘Indeterminate’ (*n* = 28). Many of these patients may have been clinically suspicious for WT. Indeterminate FNABs were either cystic contents, insufficient or demonstrated atypia or features suggestive of another salivary gland neoplasm. Repeating the FNAB and reviewing the FNAB records for those that were classified as ‘no record’ may have strengthened our findings. A further limitation of this study is the small sample size, preventing us from drawing significant conclusions from our results. However, this study has been successful in identifying specific clinical features that may be of use in the diagnosis of WT, warranting further research across multiple institutions or spanning a longer time frame.

## Conclusions

The sensitivity and PPV of FNAB consistent with WT diagnosing a final histology of WT measured in this study are high, with two false negatives. Multifocal, bilateral, incidentaloma and inferior pole location were identified as potential clinical features that may increase the diagnostic confidence for WT, strengthening the argument for conservative management in these patients. Overall, this study serves as an initial exploration into whether patient clinical features may be included with FNAB diagnosis to improve the sensitivity and PPV of a WT diagnosis. Future research projects should examine whether further subclassification of an indeterminate FNAB or repeat FNAB is helpful to improve sensitivity and PPV. A larger sample size is required in order to make definitive recommendations about observation of patients with FNAB and clinical features suggestive of WT.

## Data Availability

The datasets used and/or analysed during the current study are available from the corresponding author on reasonable request.

## Abbreviations

FNAB: Fine needle aspiration biopsy
LHSC: London Health Sciences Centre
PPV: Positive predictive value
WT: Warthin tumor
